# Preparation, Characterization and Stability Studies of Glassy Solid Dispersions of Indomethacin using PVP and Isomalt as carriers

**Published:** 2012

**Authors:** Elham Khodaverdi, Noman Khalili, Farzad Zangiabadi, Alireza Homayouni

**Affiliations:** 1 *Department of Pharmaceutics, School of Pharmacy, Mashhad University of Medical Sciences, Mashhad, Iran*; 2 *Drug Delivery Research Centre, Avicenna Institute, Mashhad University of Medical Sciences, Mashhad, Iran*

**Keywords:** Dissolution enhancement, Indomethacin, Isomalt, PVP

## Abstract

**Objective(s):**

The purpose of the present study was to use the solid dispersion (SD) technique to improve the dissolution rates of indomethacin (IMC).

**Materials and Methods:**

IMC solid dispersions in PVP K30 and isomalt (GALEN IQ 990) were prepared using the solvent evaporation technique and a hot melt method in weight ratios of 2, 10 and 30% (IMC:PVP). Solid dispersions and physical mixtures were characterized by X-ray powder diffraction (XRPD), differential scanning calorimetry (DSC) and dissolution test. Physical stability tests were also performed at different temperatures and humidity conditions.

**Results:**

The dissolution rates of all solid dispersions were faster than those of their physical mixtures. In samples containing 2% or 10% of IMC, there were no significant differences between the dissolution rates of IMC in PVP and isomalt solid dispersions, but in samples containing 30% of IMC, the dissolution rates were higher in isomalt dispersions. The XRPD analysis showed no crystalline peaks in solid dispersions, indicating that IMC was amorphous within the carrier. The DSC results showed that an interaction occurred between the drug and the carrier in PVP and isomalt dispersions. Physical stability tests at severe storage conditions showed that the dissolution rate of IMC in PVP solid dispersions decreased, while the dissolution profile of IMC in isomalt solid dispersions did not change significantly.

**Conclusion:**

It was shown that the dissolution rates of IMC in PVP and isomalt solid dispersions were substantially increased compared with their physical mixtures and pure IMC.

## Introduction

Oral drug delivery is the easiest and most comfortable way of administering drugs. The oral bioavailability of a class II drug (high permeability, low solubility) depends on its solubility or dissolution rate; therefore, an attempt to increase the dissolution rate of drugs with limited water solubility is often needed (1, 2). Several strategies are available to overcome this limitation, including salt formation of an ionizable drug, micronization, complexing drugs with cyclodextrins and the use of a co-solvent. Solid dispersions are one of the most successful methods and involve a mixture of a poorly water-soluble drug in one or more hydrophilic carriers in the solid state (3, 4). Solid dispersions can improve the dissolution rate of drugs by increasing their wettability, reducing their particle size and increasing the porosity of the final product  (4). In addition, in some solid dispersions called glassy dispersions, drugs are present in an amorphous state, which has low energy content and might better dissolve in water. The two main methods reported for the preparation of solid dispersions are the melting method and the solvent evaporation method  (4). In the melting method, solid dispersions are prepared by melting the drug within the carrier, cooling the mixture and pulverizing the final product. In this method, the miscibility of the carrier among drug molecules is the key factor to obtain an acceptable solid dispersion (5). In the solvent evaporation method, the drug and the carrier are dissolved in a common volatile solvent, and then the solvent is evaporated under a vacuum or in an oven. Finally, the resulting film is pulverized and milled (5).

Indomethacin (IMC) is a widely used non-steroidal anti-inflammatory drug (NSAID). It is used to reduce pain in osteoarthritis and rheumatoid arthritis, but because of its poor water solubility (5 μg/ml), it has a low absorption and weak bioavailability; therefore, improving their dissolution rate and/or solubility is important for the development of IMC preparations (6). 

Polymeric carriers have been the most successful carriers for solid dispersions because they can be used to prepare amorphous solid dispersions. In a study done by Dubois and Ford, the importance of the carrier to performance of the solid dispersion was illustrated in a study of 14 different drugs including IMC formulated as solid dispersions in PEG 6000. It is shown when the drug was present in a low drug/carrier ratio (2% up to 15%), the release rate depended only on the carrier and not on the drug properties (5). IMC solid dispersions in crospovidone and porous silica as carries were also investigated and the results illustrated that dissolution rates of IMC solid dispersions were substantially increased compared with IMC physical mixtures (7, 8).

 Polyvinylpyrrolidone (PVP) is water soluble and is widely used as a carrier for solid dispersions to increase the drug dissolution rate while suppressing recrystallization. Because of the high glass transition temperature (Tg) of PVP, the use of this polymer in the melt method is not possible, but its good solubility in most organic solvents makes it suitable for preparing solid dispersions by the solvent evaporation method (9).

Isomalt, a sugar alcohol, has several advantages over other polyols when formulated in pharmaceutical dosage forms. It is useful as a tablet excipient due to its properties of good taste and low hygroscopicity (10).

The high temperature stability of isomalt over its melting point (150 °C) makes this excipient suitable for preparing solid dispersions by the melt method. In addition, the high number of hydroxyl groups on its structure inhibits recrystallization in its final products (11).

The study presented here was performed to compare the suitability of isomalt and PVP as hydrophilic carriers in indomethacin solid dispersions prepared by the melt method and the solvent evaporation technique, respectively. Complete investigation of the effects of temperature and moisture on the physical stability of formulations was also examined in this study. 

## Materials and Methods

***Materials***

The indomethacin and PVP K30 were purchased from Hakim CO. IRAN. The isomalt was kindly donated by Beneo palatinit CO. Germany. The other chemicals and solvents were of analytical grade.

***Preparation of solid dispersions***

IMC solid dispersions in PVP K30 were prepared using the solvent evaporation technique in weight ratios of 2, 10 and 30% (IMC: PVP). The drug/carrier mixture was dissolved in a minimum amount of ethanol as a solvent and stirred to make a uniform dispersion. The solvent was then evaporated at 40 °C over a period of 24 hr in the oven. The obtained solid dispersions were then pulverized using a mortar and pestle and stored in desiccators at room temperature until use.

To obtain IMC solid dispersions in isomalt, the 2, 10 and 30% mixtures (IMC:isomalt) were poured into a porcelain dish while being stirred and then heated to 210 °C in a nitrogen atmosphere. After achieving a clear solution, the porcelain dish was transferred to an ice bath to solidify the mixture. Solid dispersions were then pulverized using a mortar and pestle and passed through a 100 mesh sieve. Finally, the obtained powder was stored in desiccators at room temperature until use. 

***Preparation of physical mixtures***

Physical mixtures of IMC and carriers (PVP and isomalt) in ratios of 2, 10 and 30% of drug/carrier were prepared by mechanically mixing the ingredients in a mortar and pestle for about 5 min. After mixing, the powders were stored in a desiccator at room temperature until use.

***Differential scanning calorimetry (DSC) studies***

To determine the drug/carrier interaction in IMC solid dispersions and physical mixtures, DSC studies were performed using a differential scanning calorimeter (DSC-822e Mettler-Toledo, Switzerland). Samples of the solid dispersions and physical mixtures (5 mg) were placed in sealed aluminum pans with a perforated lid. An empty pan was used as a reference. Before the experiments, the apparatus was calibrated with indium. The samples were heated at a rate of 10 °C/min from 20–400 °C in nitrogen gas. 

***X-ray powder diffraction (XRPD) studies***

The solid state of IMC in the physical mixtures and solid dispersions was evaluated using an X-ray powder diffraction method (Siemens D5000, Germany). The analysis was performed with CuK radiation at 2 varied from a 5 °C to 70 °C angular range with a voltage of 35 kV and a current of 20 mA at room temperature at a scan rate of 2°/min. 

***Dissolution studies***

Dissolution studies were performed using USP apparatus type 2 (paddle method). Samples of the IMC solid dispersions and physical mixtures equivalent to 25 mg of the drug were added to the dissolution medium (900 ml of demineralized water at a temperature of 37 °C) while stirring with a rotating paddle at 100 rpm. At selected time intervals, 5-ml samples were withdrawn, centrifuged, and analyzed for IMC at 266 nm using a UV spectrophotometer. Each test was performed in triplicate (n = 3). 

***Statistical analysis***

For comparison between the dissolution profiles of different samples, a simple independent mathematical approach that uses a difference factor (*f*_1_) and a similarity factor (*f*_2_) was proposed. The difference factor (*f*_1_) and the similarity factor (*f*_2_) were defined by the following equations:


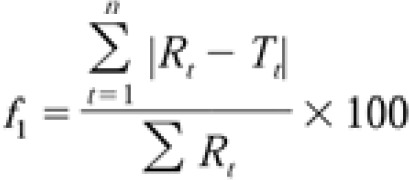



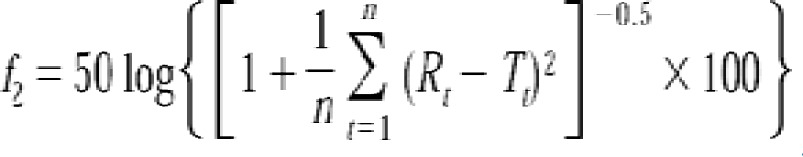


N is the number of time points, R_t_ is the percentage of the reference sample that was dissolved at the time point *t*, and T_t_ is the percentage of the test sample that was dissolved at the time point *t*.

If the *f*_1_ value was close to 0, and the *f*_2_ value was close to 100, the two curves were considered similar.

In general, if the *f*_1_ value was less than 15 (0-15), and the *f*_2_ value was greater than 50 (50-100), the dissolution profile of the two curves was considered similar (12). 

***Physical stability tests***

After preparation of the physical mixtures and solid dispersions, the samples with acceptable dissolution profiles were stored separately for 8 weeks at 25 °C and 45 °C under an RH of 70%. Every week during this time, changes in the dissolution rates of the samples were studied. To evaluate the effect of humidity on the dissolution rates of solid dispersions, the obtained powders were kept under varying RHs for 4 weeks (13). Dissolution tests were performed at the start and then at one-week intervals during this period.

## Results

***DSC studies***

**Figure 1:**
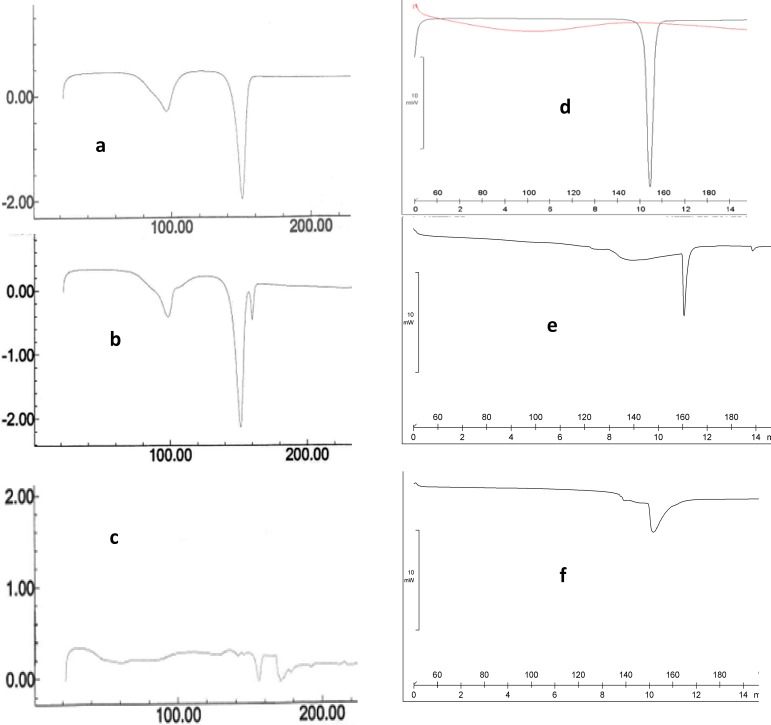
DSC thermograms of (a) pure isomalt, (b) a physical mixture of IMC (10%)-isomalt, (c) a solid dispersion of IMC (10%)-isomalt, (d) pure IMC and PVP, (e) a physical mixture of IMC (10%)-PVP and (f) a solid dispersion of IMC (10%)-PVP (f).

DSC thermograms of the pure IMC and PVP, IMC physical mixtures and IMC solid dispersions at a 10% drug/carrier ratio are shown in Figure 1.

As shown in Figure 1a, the first peak in the DSC thermogram of pure isomalt was related to its Tg, and the second peak (near 150 °C) was considered its melting point. The melting point of IMC was 160°C indicating the presence of polymorph II, as shown in Figure 1d. 

***X-ray powder diffraction (XRPD) studies***

**Figure 2:**
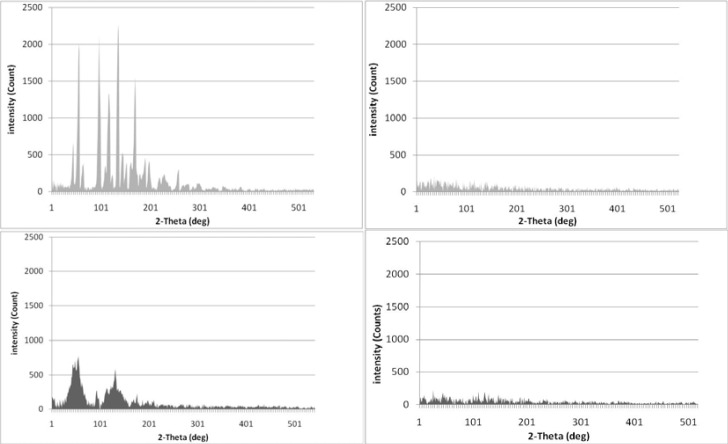
The XRPD spectra of, (a) pure IMC, (b) PVP, (c) a physical mixture of IMC-PVP and (d) a solid dispersion of IMC-PVP

The XRPD spectra of pure IMC and PVP, a 10% IMC physical mixture and a 10% IMC solid dispersion in PVP exhibited essentially different diffraction patterns, shown in Figure 2. 

***Dissolution studies***

**Figure 3:**
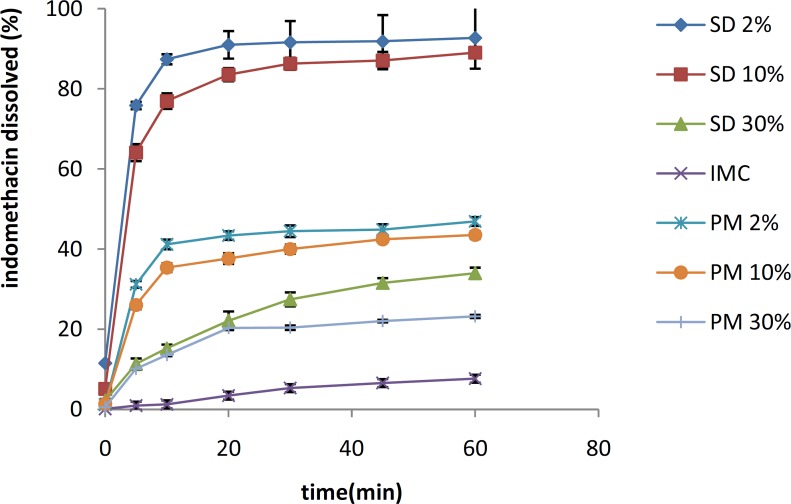
The dissolution profiles of pure IMC, IMC/PVP solid dispersions and IMC/PVP physical mixtures at different drug/carrier ratios. (SD: solid dispersion. PM: physical mixture)

**Figure 4:**
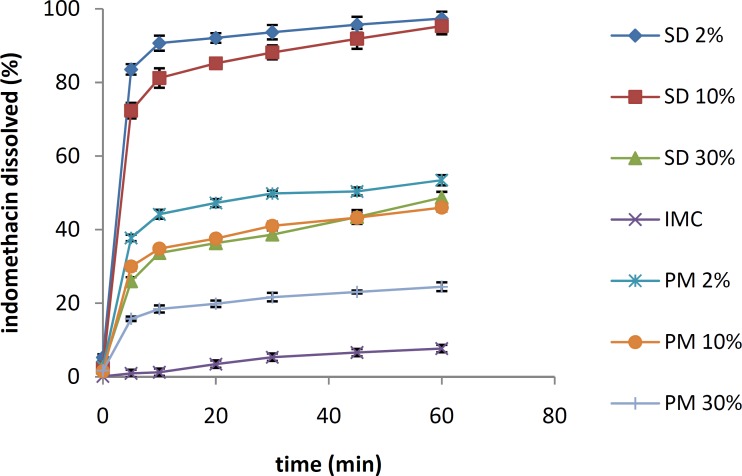
The dissolution profiles of pure IMC, IMC/isomalt solid dispersions and IMC/isomalt physical mixtures at different drug/carrier ratios. (SD: solid dispersion. PM: physical mixture)

The dissolution patterns of pure IMC, IMC physical mixtures and IMC solid dispersions prepared with PVP and isomalt are shown in Figure 3 and Figure 4, respectively. It is clear that all solid dispersions had higher dissolution rates compared with the pure IMC and the physical mixtures of the same drug percentage.

The maximum dissolution rate of the IMC/isomalt solid dispersions was found in the formulation containing 2% of the drug. With an increase in the drug ratio to 10%, a negligible decrease in dissolution rate was observed (*f*_1_=8.19, *f*_2_=55.14), while an increase to 30% dramatically decreased the rate of dissolution (*f*_1_=73.29, *f*_2_=9.37). 

**Figure 5:**
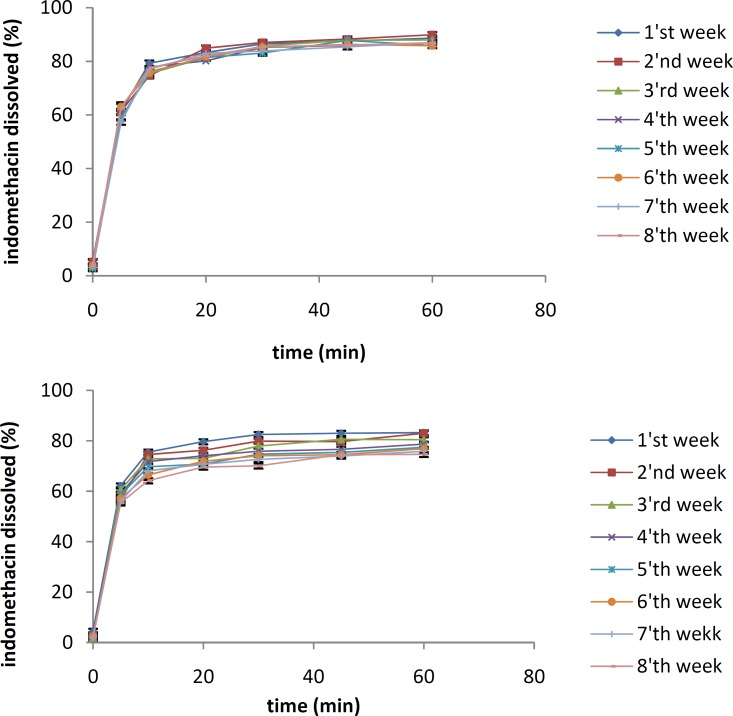
The dissolution rates of 10% IMC-PVP solid dispersion samples after storing at 25 °C (top) and 45 °C (bottom) for 8 weeks

As shown in Figure 4 and 5, the dissolution rates of IMC in its physical mixtures were faster than that of pure IMC, but they were much slower than the IMC solid dispersions of the same drug/carrier ratio. In the dissolution profile of IMC/PVP solid dispersions, similar data were obtained. Similar to the IMC/isomalt solid dispersions, the highest dissolution rate was observed in the IMC/PVP solid dispersions containing 2% and 10% of the drug, and no significant difference was observed between these two formulations (*f*_1_=7.05, *f*_2_=56.87). The dissolution rate of the IMC/PVP solid dispersion containing 30% of the drug was dramatically decreased, and it became even slower than that of the IMC/PVP physical mixtures containing 2% and 10% of the drug (*f*_1_=58.99, *f*_2_=13.20).

***Physical stability tests***

**Figure 6:**
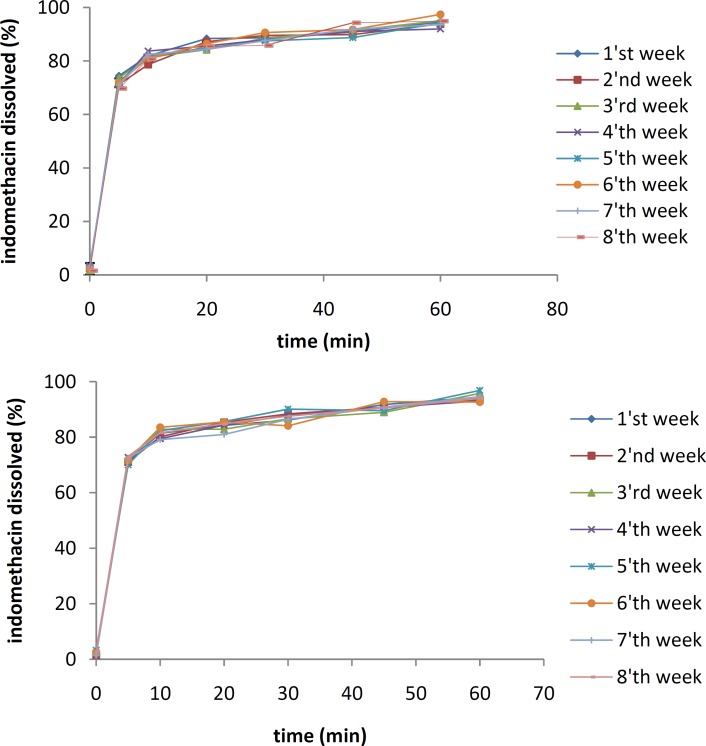
The dissolution rates of 10% IMC-isomalt solid dispersion samples after storing at 25 °C (top) and 45 °C (bottom) for 8 weeks

The effects of temperature and moisture on the dissolution profile of the IMC solid dispersion containing 10% of the drug are shown in Figure 5 and 6, respectively. The samples were stored separately for 8 weeks at 25 °C and 45 °C under an RH of 70%.

The dissolution studies indicated that after 8 weeks, there were no significant differences between the dissolution profiles of IMC/PVP solid dispersions at 25 °C (*f*_1_=1.32, *f*_2_=88.69). However, a significant reduction in the IMC dissolution rate was shown after 8 weeks for the samples at 45 °C (*f*_1_=16.17, *f*_2_=43.71). All samples were kept at an RH of 70%.

As shown in Figure 8, no significant difference in the dissolution profile of the IMC/isomalt solid dispersion was observed after 8 weeks not only for the samples at 25 °C (*f*_1_=4.93, *f*_2_=65.63), but also for the ones at 45 °C (*f*_1_=5.06, *f*_2_=65.16). 

**Figure 7:**
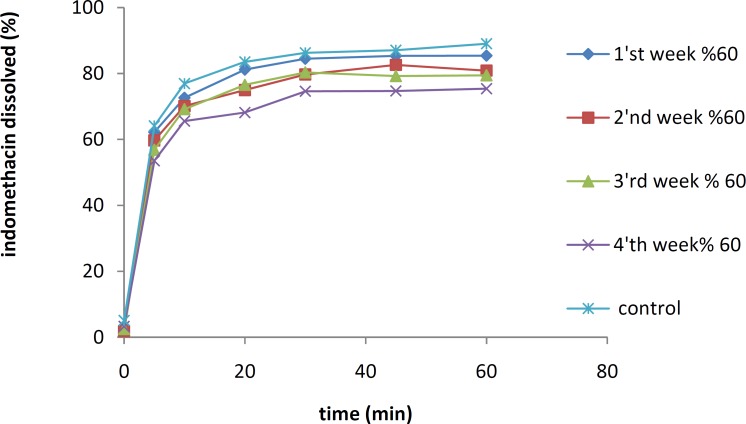
The dissolution rates of a 10% IMC-PVP solid dispersion after storing at 25 °C and RH of 60%

**Figure 8:**
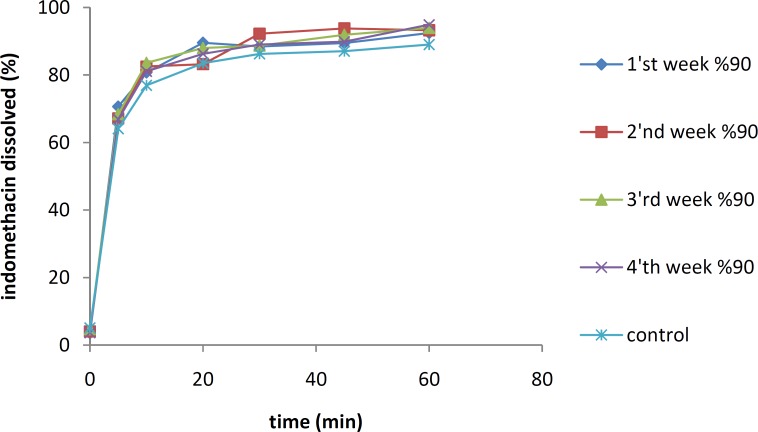
The dissolution rates of a 10% IMC-PVP solid dispersion after storing at 25 °C and RH of 90%

The stabilities of IMC/PVP solid dispersions and IMC/isomalt solid dispersions containing 10% of the drug were also investigated at different RH conditions over a period of one month initially (control), and then at one-week intervals. As shown in Figure 7 and Figure 8, the dissolution rate of the IMC/PVP solid dispersion was decreased at an RH of 60% while the reduction in the dissolution rate at RHs of 70 and 80% were not considered significant. To decrease the numbers of figures, data for RHs of 70 and 80% were not shown. As the RH mounted to 90%, an increase in the IMC dissolution rate was observed. 

**Figure 9:**
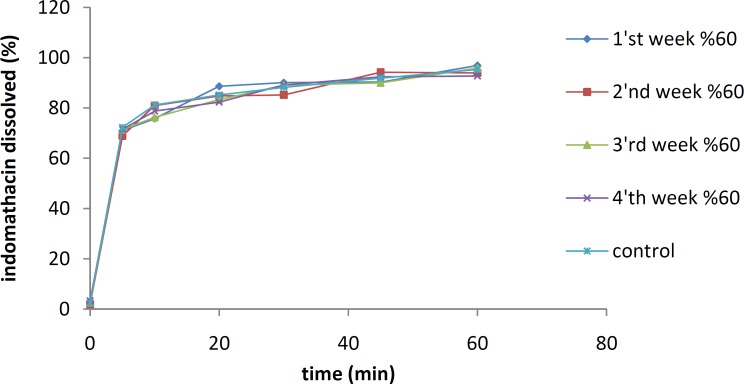
The dissolution rates of a 10% IMC-isomalt solid dispersion after storing at 25 °C and RH of 60%

**Figure 10:**
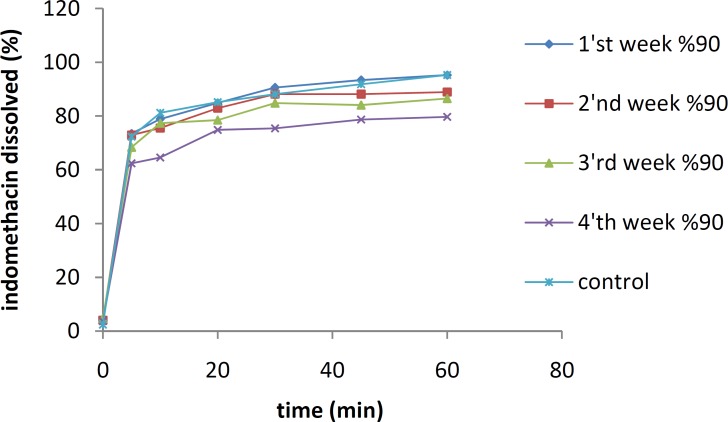
The dissolution rates of a 10% IMC-isomalt solid dispersion after storing at 25°C and RH of 90%

The effects of different humidity conditions on the dissolution rates of IMC-isomalt solid dispersions are shown in Figure 9 and 10. The data illustrated no significant difference in the IMC dissolution rate at RH of 60%. However, for the samples stored at an RH of 90%, the drug-dissolution rate was decreased at the 4th week compared with the beginning (control) due to the recrystallization of amorphous IMC (_1_=15.20, *f*_2_=43.83).

**Figure 11:**
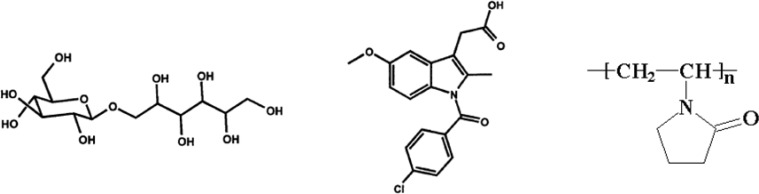
The chemical structures of (a) isomalt, (b) IMC (c) and PVP

## Discussion

DSC can be used to determine if any changes in the crystal state or the amorphous form of the drug have been accrued. As shown in Figure 1d, the melting point of IMC was 160 °C indicating the presence of polymorph II. 

This method can also determine the interaction between IMC and its carriers. In the thermogram of a 10% IMC solid dispersion in isomalt, no sharp peaks was observed, which could be due to the formation of an amorphous state of ingredients in the solid dispersion. As shown in Figure 1, the same result could be reached for the PVP samples. In the DSC thermogram of a 10% IMC solid dispersion in PVP, there is no sharp peak similar to the one observed in its physical mixture. In addition, the melting point of IMC was shifted from 160 °C to 150 °C in this thermogram. This reduction in the IMC melting point might be attributed to the presence of an amorphous state of drug in its solid dispersion.

By looking at the chemical structure of PVP and isomalt (Figure 11), it can be concluded that these two materials can interact with IMC via hydrogen bonding. This interaction is important to increase the physical stability of solid dispersions over time. 

Langer *et al* demonstrated that the presence of isomalt in acyclovir and theophylline solid dispersions caused changes in the crystalline structures of the drugs and formation of their amorphous states (11). Wang *et al* prepared an IMC solid dispersion in PVP and evaluated the physical states of the ingredients in the samples via differential scanning calorimetry. DSC studies showed significant changes in the melting point of IMC, which could indicate an interaction between PVP and IMC (6). 

The XRPD spectra of a 10% IMC solid dispersion also indicated an amorphous state of drug present in the formulation, compared with a crystalline form in the formulations of pure IMC and an IMC physical mixture. The amorphous state of IMC in a solid dispersion was also confirmed by DSC in the previous section. Fujii *et al* evaluated solid dispersions of IMC in crospovidone. X-ray powder diffraction analysis did not show any sharp peaks in the solid dispersions. Their data illustrated an amorphous structure of the drug in their samples (7).

The results of dissolution studies indicated that all of the solid dispersions had higher dissolution rates compared to the pure IMC and the physical mixtures of the same drug percentage.

Some of these increases in the dissolution rate of IMC solid dispersions were attributed to a decrease in the particle size of IMC in the related formulations. Moreover, in the IMC solid dispersions, the drug particles were in close contact with a hydrophilic carrier (PVP or isomalt), which caused better wettability of IMC and, consequently, a higher drug-dissolution rate (14). Another reason for the improved dissolution of IMC in its solid dispersions was the amorphous state of IMC in these formulations. The amorphous state of a drug always has a higher level of energy and subsequently, a higher dissolution rate compared with the crystalline state in its physical mixtures (5).

An increase in the drug/carrier ratio causes incomplete coverage of the drug molecules by the hydrophilic carrier, which causes a reduction in the wettability and, consequently, a decrease in the drug dissolution rate. In a study performed by Kapsi *et al*, successful solubilization of itraconazole was achieved using solid solution techniques. The solid solutions with lower drug concentrations gave faster dissolution rates, and drug dissolution improved considerably with an increase in the molecular weight of PEG (15). 

In a comparison between IMC/PVP solid dispersions and IMC/isomalt solid dispersions in a specified drug/carrier ratio, no significant differences were observed among systems containing 2% (*f*_1_=4.09, *f*_2_=67.70) and 10% of the drug (*f*_1_=5.26, *f*_2_=64.33), while the IMC/isomalt solid dispersion containing 30% of the drug showed a higher dissolution rate compared with the 30% IMC/PVP solid dispersion (*f*_1_=37.53, *f*_2_=72.07). 

In a study performed by Langer *et al*, four sugar alcohols, such as isomalt, with different drug models were investigated for the formation of glassy solid dispersions using the melting method. It was concluded that due to the relatively high Tg of isomalt (61.5 ◦C) and the absence of transformations after melting because of the number of hydroxyl groups on its structure, this sugar alcohol seemed to be best suited as a carrier for glassy solid solutions. However, in that study, the dissolution rates of the solid dispersions containing isomalt and their stabilities at different humidity values and temperatures were not investigated (11).

The results of physical stability tests done at 25 °C and 45 °C indicate that an increase in temperature triggers molecular mobility of the drug and the carriers, which might accelerate recrystallization of the amorphous IMC. When the amorphous state of IMC in solid dispersions is converted to the crystalline form, a reduction in drug dissolution rate occurs (16, 17). 

The IMC/isomalt solid dispersions showed better physical stability than the IMC/PVP solid dispersions under high temperature conditions. This can be attributed to the large number of hydroxyl groups on the molecule of isomalt, which causes a higher probability of an interaction between the drug and the carrier. More interaction increases the stability of an amorphous form of IMC and inhibits its crystal growth (11). The results of physical stability tests at different RHs indicated that the dissolution rate of the IMC/PVP solid dispersion was decreased at an RH of 60%. This effect may be due to the absorption of water by PVP and the recrystallization of IMC. 

The IMC dissolution rates at RHs of 70, 80 and 90% were expected to be further reduced due to the faster recrystallization of the drug in higher moisture, but the observation was different. At RHs of 70 and 80%, the changes in drug dissolution rate were not significant. It seems that water molecules play the role of plasticizer in formulations and cause a decrease in the Tg of the polymer. Upon a reduction in Tg, the polymer chain was able to surround the drug molecules and prevent the process of IMC recrystallization. As the RH increased up to 90%, the effect of the water molecules as a plasticizer became dominant over the recrystallization of IMC, and an increase in the IMC dissolution rate was observed. The *f*_1_ and *f*_2_ factors were 18.94 and 39.51, respectively, for the curves of the 4^th^ week of the dissolution profiles at 60 and 90% humidity. This result illustrated a significant difference between these two curves. Physical stability tests indicated that isomalt solid dispersion samples showed better physical stability in various levels of humidity compared with PVP K30 dispersion samples. This effect can be attributed to the large number of hydroxyl groups on the structure of isomalt, which can form hydrogen bonds with IMC to increase the physical stability of the amorphous drug. 

## Conclusion

Preparation of solid dispersions is one of the most useful processes for improving the solubility of poorly water-soluble drugs. In this study, the dissolution rate of IMC was determined after dispersion in PVP K30 and isomalt with different ratios of drug to carrier (2, 10 or 30%). XRPD and DSC studies revealed that IMC was present in an amorphous state in the solid dispersions as a result of an interaction between the drug and the carrier in these systems. An increase in the dissolution rate of IMC/PVP and IMC/isomalt solid dispersions was observed compared with their physical mixtures and pure IMC. This increase could be due to better wettability of IMC within these hydrophilic carriers and/or a result of the amorphous form of the drug in IMC solid dispersions. IMC/isomalt solid dispersions showed better physical stability compared with IMC/PVP solid dispersions under higher moisture and higher temperature conditions. This result may be attributed to the large number of hydroxyl groups on the isomalt molecules, which causes better interaction between the drug and the carrier and consequently, better physical stability of the amorphous form of the drug in IMC/isomalt systems compared with IMC/PVP solid dispersions. 
